# Probiotic and Dietary Supplements Intervention in Age-Related Neurodegenerative Disorders

**DOI:** 10.3390/microorganisms14020290

**Published:** 2026-01-27

**Authors:** Carolina Beatrice D’Anniballe De Salles, Santosh Kumar Prajapati, Dhananjay Yadav, Joell Rennar, Andrea Marcano-Rodriguez, Hariom Yadav, Shalini Jain

**Affiliations:** 1 USF Center for Microbiome Research, Microbiomes Institute, University of South Florida Morsani College of Medicine, Tampa, FL 33613, USA; carolinabeatrice@usf.edu (C.B.D.D.S.); prajapati11@usf.edu (S.K.P.); dhananjay11@usf.edu (D.Y.); joellr@usf.edu (J.R.); acm11@usf.edu (A.M.-R.); hyadav@usf.edu (H.Y.); 2 Department of Neurosurgery, Brain and Spine, Center of Excellence for Aging and Brain Repair, University of South Florida Morsani College of Medicine, Tampa, FL 33612, USA

**Keywords:** neurodegenerative diseases, gut–brain axis, probiotics, dietary supplements, nutritional interventions, cognitive impairment

## Abstract

Age-related neurodegenerative disorders, including Alzheimer’s disease, Parkinson’s disease, and mild cognitive impairment, represent a growing global health challenge. The present medicines offer only symptomatic alleviation with poor disease-modifying efficacy. Increasing data suggests that the gut–brain axis and dietary health are measurable contributions to cognitive impairment as we age. This review first focused on the mechanistic link between gut dysbiosis and neurodegeneration. Furthermore, the review discusses preclinical and clinical research that show how probiotics and dietary supplements improve brain function in the elderly using supplemental therapy methods. It also indicates that randomized clinical studies and meta-analyses suggest that probiotics and particular nutritional supplements provide modest but consistent cognitive advantages, which are most noticeable when patients receive therapy at the initial stage of their disease development. These advantages might originate from the combined impact of gut microbiota, immunological signaling, and neuroprotective pathways, rather than specific targeted approaches. Thus, the current review highlights the reports, suggesting that probiotics and dietary supplements might be effective and safe therapies for age-related neurodegeneration.

## 1. Introduction

The worldwide health crisis from neurodegenerative diseases like Alzheimer’s disease (AD), Parkinson’s disease (PD), and mild cognitive impairment (MCI), affect older adults has become more severe [1,2,3]. The worldwide dementia case numbers will reach 131 million during 2050 according to projections while AD will continue to cause 60–80% of all diagnosed dementia cases [4,5]. The number of people who develop PD keeps rising which creates major physical and emotional and financial challenges for patients and their caregivers and for healthcare organizations [6,7]. Currently available treatments for these diseases are limited and primarily focus on symptomatic management [8]. Further, these treatments do not stop disease progression and are often associated with adverse effects. Therefore, there is a need to identify alternative therapeutic strategies with disease-modifying potential and minimal side effects [5,9].

In recent years, several preclinical and clinical studies have represented the effectiveness of probiotics and dietary supplements in the management of neurodegenerative disorders [10,11,12,,13]. Therefore, the current review elaborated the evidence that demonstrates probiotics and dietary supplements as a treatment’s strategy for neurodegenerative diseases.

The gut microbiome undergoes sequential changes which result in dysbiosis during aging because it loses its beneficial commensal bacteria while pathogenic bacteria multiply [14]. As a result, there is an increase in gut permeability, oxidative stress, and inflammation, commonly known as an inflammaging [10,15,16,17]. The increased intestinal permeability facilitates the migration of pathobionts and inflammatory mediators into the systemic circulation. Circulating inflammatory cytokines can subsequently reach the brain, facilitating neuroinflammation, amyloid-β (Aβ) and tau pathology [7,18,19,20,21].

Furthermore, alteration in gut microbiota and dietary create new inflammatory conditions which then modify neuroimmune signaling, synaptic plasticity and metabolic function [15,18,22,23]. The microbial changes which affect both microbial community structure and metabolic output of short-chain fatty acids (SCFAs) and tryptophan-derived indoles lead to microglial activation and affect neurotransmitter stability and blood–brain barrier (BBB) function [21,24,25,26]. These interactions underscore the central role of the gut–brain axis in maintaining cognitive health during aging.

Researcher now understand that nutritional intervention serve as vital protective measures which help maintain mental functions during aging [27,28]. For example, probiotics restore gut integrity and immune responses by producing neuroactive metabolites such as SCFAs [16,19,20]. Therefore, the prevention of age-related cognitive decline needs to explore gut dysbiosis and nutritional deficiencies because these conditions provide effective and affordable methods to delay or prevent neurodegeneration. Research evidence demonstrates that probiotics and dietary supplements present a safe treatment option which healthcare providers can use to support existing medications while protecting brain cells [19,28,29].

**Figure 1:**
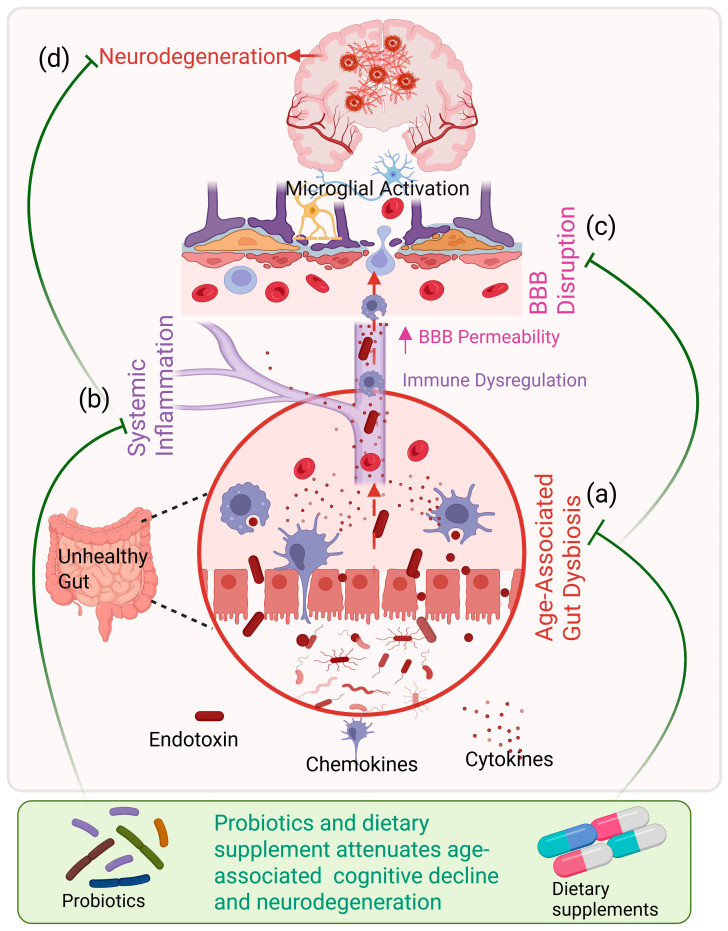
Unhealthy aging–associated gut–brain axis dysfunction and its modulation by nutritional interventions. During unhealthy aging, reduced microbial diversity promotes gut dysbiosis (a), leading to increased intestinal permeability and translocation of endotoxins. This triggers systemic inflammation characterized by elevated circulating cytokines and chemokines (b). Persistent inflammatory signaling compromises blood–brain barrier integrity and induces glial cell activation (c), ultimately contributing to neurodegeneration and cognitive decline (d). Probiotics and dietary supplements modulate gut microbiota composition and attenuate age-associated gut dysbiosis, systemic inflammation, BBB permeability and neurodegenerative risk.

Thus, this review integrates available preclinical and clinical evidence to evaluate the therapeutic potential of probiotics and dietary supplements in AD, PD, and MCI. It highlights how these interventions collectively support brain health in older adults by modulating microbial composition, immune function, and nutritional status (Figure 1). Figure 1 illustrates the key links between aging-associated alterations in the gut microbiome and the neuroprotective effects of probiotics and nutritional interventions mediated through the gut–systemic–brain axis.

## 2. Methods

A narrative approach was adopted to enable integrative evaluation of heterogeneous evidence, including mechanistic studies, animal models, and human clinical investigations related to the gut–brain–immune axis. Further, human randomized controlled trials were prioritized when discussing clinical efficacy and cognitive outcomes. Relevant literature was identified through searches of PubMed/MEDLINE, Scopus, and Web of Science, with additional studies retrieved from Google Scholar and by manual screening of reference lists from key reviews and influential primary articles. Studies were included if they were peer-reviewed, published in English, and examined probiotics and/or dietary supplements in relation to cognitive outcomes, neurodegeneration, neuroinflammation, or gut–brain axis mechanisms in aging, AD, PD, and MCI.

## 3. Mechanistic Interface: Aging, Gut Dysbiosis, and Neurodegeneration

The gut–brain axis (GBA) is a two-way communication network that connects the central nervous system (CNS) with the enteric nervous system (ENS), immune signals, and microbial metabolites [30,31]. The neural pathways mediated by the vagus nerve represent a major route of communication between the gut and the brain [32]. Age-related ENS degeneration and reduced vagal tone may impair signaling, affecting gastrointestinal motility and central regulation of inflammation [10]. Further, gut dysbiosis stimulates immune responses that increase circulating pro-inflammatory cytokines, such as IL-6 and TNF-α. These cytokines activate microglia in the brain and contribute to neuroinflammation in AD and PD [16,33]. In addition to immune dysregulation the hypothalamic–pituitary–adrenal (HPA) axis becomes dysregulated during aging according to studies [34,35]. Long term activation of the HPA axis results in increased cortisol release, which exacerbates neuroinflammation and increases vulnerability to neurodegeneration in disorders such as AD and PD [35]. Moreover, the metabolic dysregulation represents another critical pathway linking gut dysfunction to the progression of neurodegenerative diseases [36]. Further, age-related alterations in microbial metabolism and host energy homeostasis impair SCFA production, mitochondrial function, and glucose and lipid metabolism, leading to increased oxidative stress and sustained neuroinflammation [22,37,38]. These metabolic disturbances compromise neuronal energy supply, disrupt blood–brain barrier (BBB) integrity, and exacerbate protein misfolding and synaptic dysfunction in AD and PD. Aging reduces SCFA production while altering tryptophan metabolism, leading to decreased serotonin levels and increased production of neurotoxic metabolites such as quinolinic acid [7]. Therefore, gut dysbiosis, inflammaging, and altered GBA accelerate brain damage and cognitive decline. Thus, using probiotics and dietary supplements could help restore balance to these linked pathways. Additionally, studies have shown there was an reduction in health-promoting commensals bacteria such as *Bifidobacterium* and *Faecalibacterium*, and an increase in potentially pathogenic taxa (*Enterobacteriaceae*) [10,16]. Moreover, it is demonstrated that older adults with lower levels of *Bifidobacterium* were more likely to show mild cognitive impairment [39]. Furthermore, the Firmicutes/Bacteroidetes (F/B) ratio was higher in the demented patients than the non-demented patients [39]. Other studies reported different microbial signatures in AD patients, characterized by a loss of anti-inflammatory mediators while an increase in pro-inflammatory groups [40]. These data support the hypothesis that the composition of the gut microbiota serves as a marker and influences cognitive aging. Together, these strategies may help counter the negative effects of age-related dysbiosis.

Targeting the GBA with probiotics and dietary supplements is a promising way to reduce age-related neurodegeneration. Probiotics help restore a healthy gut by improving microbial diversity, slowing the growth of harmful bacteria, and producing SCFAs that have anti-inflammatory and neuroprotective effects [14,16,19]. According to the FAO/WHO definition, probiotics are live microorganisms that provide health benefits to the host when consumed in the right amounts [41]. The most studied genera are Lactobacillus (e.g., *L. acidophilus*, *L. plantarum*, *L. casei*) and *Bifidobacterium* (e.g., *B. longum*, *B. breve*, *B. bifidum*) [42]. However, other strains, including *Streptococcus thermophilus*, *Bacillus subtilis*, *Clostridium butyricum*, and *Escherichia coli Nissle 1917* have also been examined for their therapeutic benefits [16,19,43]. These strains can survive the gastrointestinal tract, stick to intestinal cells, and affect the host’s immunity and metabolism. In addition to these well-known types, new probiotics like *Akkermansia muciniphila* and *Faecalibacterium prausnitzii* are gaining interest for how they influence inflammation and support gut health [41,44]. These probiotics modulate host immunity, metabolism, and neurotransmitter production, including serotonin and GABA, thereby influencing cognition and mood [45]. *Lactobacillus* and *Bifidobacterium*, help rebalance the microbiota and boost the production of mood-regulating and cognition-enhancing neurotransmitters like serotonin and GABA [16,45]. Moreover, probiotics improve the BBB integrity. This reduces the entry of harmful cytokines and toxins into the CNS [46]. Both clinical and preclinical studies support their ability to lower systemic inflammation, increase neurotrophic factors, and enhance cognitive performance [19,29].

Moreover, with age, microbially derived metabolites entering the circulation are also recognized as key messengers linking gut status to systemic and brain physiology. Among these substances are the SCFAs (acetate, propionate, butyrate) produced during fiber fermentation and tryptophan-derived indoles. SCFAs can shift immune tone, enhance epithelial and BBB tight junctions, and can reach the brain to influence microglial state [24,47]. In neurodegenerative models, probiotic or multi-strain supplementation increases circulating SCFAs and associates with reduced peripheral cytokines [18]. Indole derivatives, particularly indole-3-propionic acid, exert antioxidant effects and suppress astrocytic and microglial inflammation via aryl hydrocarbon receptor signaling, thereby protecting BBB integrity and attenuating neuroinflammatory cascades linked to cognitive decline [25,26,48,49].

Furthermore, SCFAs are key regulators of peripheral immune homeostasis and immunometabolism. Butyrate and propionate promote regulatory T-cell (Treg) differentiation through histone deacetylase inhibition at the *Foxp3* locus, fostering immune tolerance and mitigating inflammaging [47,50,51]. While SCFAs support Treg expansion under homeostatic conditions, they also enhance effector T-cell metabolism and cytokine production during immune activation, underscoring their context-dependent immunomodulatory role [52]. Probiotic-derived metabolites further contribute to immune balance by suppressing Th17 responses and lowering systemic IL-6 and TNF-α levels [19,51,53]. Treg/Th17 balance is relevant in aging, where chronic low-grade inflammation (inflammaging) can increase the risk of neurodegeneration [15].

At the same time, dietary supplements like prebiotics, polyphenols, and fibers can feed beneficial bacteria, boost SCFA production, and affect the gut–brain connection [28]. Additionally, diets rich in polyphenols have been shown to change the microbiota toward healthier profiles while also lowering oxidative stress [54]. The use of multi-nutrient formulations, such as Souvenaid, has also been studied in clinical trials. These showed modest improvements in memory performance in patients with AD [55]. Dietary supplements-such as omega-3 polyunsaturated fatty acids (PUFAs), B-complex vitamins, vitamin D, vitamin E, polyphenols, and prebiotics-support neuroprotection through anti-inflammatory, antioxidant, and microbiota-modulating mechanisms [27,28,47]. PUFAs, especially docosahexaenoic acid (DHA) and eicosatetraenoic acid (EPA), which are abundant in nerve cells, support synaptic flexibility and lower neuroinflammation [28]. They enter the neuronal membrane, help maintain synaptic plasticity, and reduce microglial hyperactivity [28]. Polyphenols are plant-derived compounds that include flavonoids (from berries, tea, cocoa) and curcumin that have antioxidant effects, modulate intracellular signaling pathways (e.g., MAPK, NF-κB), and alter gut microbiota composition [28]. Prebiotics are non-digestible dietary fibers (e.g., inulin, fructo-oligosaccharides) that selectively stimulate the growth and activity of beneficial bacteria, promoting SCFA production and gut–brain axis function [10]. Clinical and mechanistic studies show that B vitamins reduce plasma homocysteine, a known risk factor for cognitive decline, thereby supporting neuroprotection [56]. Omega-3 fatty acids not only reduce inflammation but also promote hippocampal synaptogenesis and neurogenesis [57]. Among polyphenols, resveratrol has been demonstrated to improve hippocampal functional connectivity and memory in older adults [58]. Prebiotic supplementation, by enhancing SCFA levels, plays a role in the maturation and function of microglia, further linking the microbiome to neuroprotection [24]. Collectively, these interventions act synergistically to support gut barrier integrity, immune balance, and cognitive resilience in aging.

Dietary supplements also shape immune and metabolic signaling vitamin D promotes Treg polarization and suppresses Th17 activity [59,60,61]. However, omega-3 PUFAs are converted into specialized pro-resolving mediators (SPMs) that actively resolve inflammation, enhance macrophage efferocytosis, and reduce microglial activation [62,63,64]. SPMs actively resolve inflammation, decrease oxidative stress, and stimulate debris phagocytosis [64]. Clinical studies link higher omega-3 status with reduced systemic inflammation and improved cognition in older adults [65,66]. Therefore, these interventions promote neuroprotection through enhanced antioxidant defenses, regulation of neurotransmitter systems, and support of neurotrophic factors [19,28]. As such, dietary and microbial strategies represent low-cost, accessible adjuncts that could complement existing therapies and potentially delay the progression of age-related neurodegenerative disorders. Together, probiotics and targeted nutritional interventions strengthen barrier integrity, regulate cytokine profiles, improve redox balance, and support microglial homeostasis, collectively reducing inflammaging and lowering the risk of age-related cognitive decline [29,54,67]. Overall, these findings support a multidomain approach, where microbiota modulation through probiotics and targeted supplementation may help mitigate inflammaging and preserve cognitive health in the elderly.

## 4. Interventions Based on Different Neurodegenerative Diseases

**Figure 2:**
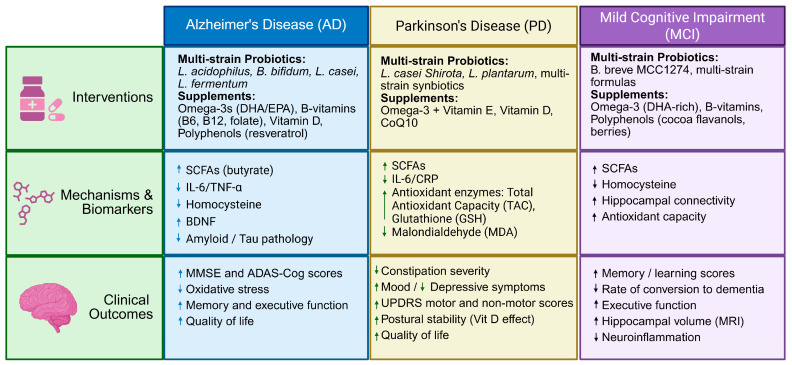
Overview of probiotic and dietary supplement interventions in Alzheimer’s disease (AD), Parkinson’s disease (PD), and mild cognitive impairment (MCI). Across conditions, probiotics and selected supplements are associated with modulation of gut microbiota, inflammatory and metabolic biomarkers, and neuroprotective pathways. Arrow ↑ means increase, and ↓ means decrease.

Figure 2 offers a summary comparing probiotic and dietary supplement interventions. It details the mechanisms, biomarkers, and clinical outcomes related to AD, PD, and mild MCI. Furthermore, Table 1 represents clinical trials evaluating probiotics and dietary supplements in age-related neurodegenerative disorders.

### 4.1. Alzheimer’s Disease (AD)

AD is neuropathologically characterized by extracellular amyloid-β (Aβ) deposition, tau hyperphosphorylation with neurofibrillary tangle formation, synaptic and neuronal loss, oxidative stress, and chronic neuroinflammation [68,69,70]. Increasing evidence indicates that these hallmark processes are modulated by systemic metabolic dysfunction, immune aging, and gut microbiome dysbiosis.

#### 4.1.1. Preclinical Evidence

Preclinical studies consistently demonstrate that microbiota-targeted nutritional interventions can attenuate AD-related pathology. In 3xTg-AD mice, supplementation with *Lactobacillus plantarum* KY1032 and *L. curvatus* HY7601 reduced microglial activation, preserved hippocampal neuronal integrity, and improved spatial memory [29]. In tauopathy models, probiotic administration decreased neuroinflammation and inhibited glycogen synthase kinase-3β (GSK-3β) activity, leading to reduced tau hyperphosphorylation and improved cognitive performance [46]. These effects have been linked to increased circulating SCFAs, which enhance BBB integrity and upregulate neurotrophic factors such as brain-derived neurotrophic factor (BDNF) [16,24,45,51]. Beyond probiotics, dietary bioactives have demonstrated complementary benefits. Polyphenols including resveratrol and curcumin reduced amyloid burden, oxidative stress, and neuroinflammation in APP/PS1 mouse models [71,72]. While omega-3 fatty acids enhanced synaptic resilience and increased BDNF expression [73,74]. Collectively, preclinical findings support a disease-modifying potential of microbiota-modulating and nutritional strategies in early AD-like pathology.

#### 4.1.2. Clinical Evidence

Clinical studies provide converging evidence that probiotics and targeted nutritional supplements exert measurable benefits in early disease stage of AD. In a landmark randomized controlled trial, Akbari et al. reported that 12 weeks of probiotic milk supplementation containing *Lactobacillus acidophilus*, *L. casei*, *Bifidobacterium bifidum*, and *L. fermentum* significantly improved Mini-Mental State Examination (MMSE) scores in AD patients [53]. Similarly, Tamtaji et al. demonstrated that probiotic and selenium co-supplementation improved MMSE scores, enhanced antioxidant enzyme activity, and reduced inflammatory cytokines (IL-6, TNF-α) [75]. In individuals with mild cognitive impairment, supplementation with *Bifidobacterium breve* MCC1274 improved memory and visuospatial performance while lowering C-reactive protein and IL-1β levels [76]. Nutritional interventions have shown parallel benefits. Omega-3 fatty acid supplementation improved cognitive outcomes in individuals with MCI and AD [65,66]. Further, vitamin D supplementation has been associated with improved immune polarization through induction of regulatory T cells and suppression of Th17 responses [61]. However, B-complex vitamins (B6, B12, folate) reduce homocysteine-associated neurotoxicity linked to hippocampal atrophy and cognitive decline [56]. Polyphenols such as resveratrol improved memory performance and hippocampal functional connectivity in older adults [58]. Moreover, multi-nutrient formulations, including Souvenaid, which combines omega-3 fatty acids, uridine, choline, and cofactors, consistently improved memory performance in patients with prodromal and mild AD, with diminishing effects in advanced stages [55].

Meta-analyses further strengthen the clinical evidence base. Naomi et al. analyzed 23 randomized controlled trials and reported small but significant improvements in MMSE scores, alongside favorable effects on insulin metabolism and oxidative stress biomarkers [19]. Additionally, Tripathi et al. observed that cognitive benefits were most pronounced in memory domains and were greater with longer intervention duration and multi-strain probiotic formulations [77]. An umbrella meta-analysis by Xiao et al. confirmed reproducible improvements in MMSE and ADAS-Cog scores, as well as cardiometabolic benefits such as reduced insulin resistance and improved lipid profiles, concluding that probiotics represent a low-risk adjunctive intervention [78]. Additional pooled analyses emphasized gut microbiota restoration, attenuation of systemic inflammation, improved glucose metabolism, and reduced oxidative stress as convergent mechanisms underlying clinical benefit [79,80].

In summary, evidence from both preclinical and clinical studies indicates that probiotics and targeted nutritional supplements exert modest but reproducible benefits in AD, with the strongest effects observed in prodromal and early disease stages. These interventions appear most effective as adjunctive strategies, complementing existing therapies by modulating systemic inflammation, metabolic regulation, and microbiota-dependent signaling pathways relevant to AD pathogenesis.

### 4.2. Parkinson’s Disease (PD)

PD is characterized by the progressive loss of dopaminergic neurons in the substantia nigra [81]. This neuronal degeneration leads to classical motor symptoms such as tremor, rigidity, and bradykinesia. Growing evidence supports the gut hypothesis of PD, suggesting that α-synuclein pathology may begin in the gut. From the enteric nervous system, this pathology is thought to spread to the brain through the vagus nerve [22,82,83].

#### 4.2.1. Preclinical Evidence

Animal models of PD provide mechanistic support for microbiota-targeted and nutritional interventions. In rotenone-induced PD rat models, *Lactobacillus plantarum* supplementation improved motor performance and significantly reduced oxidative stress and neuronal damage [84]. Further, Xie and Prasad demonstrated that treatment with *Lacticaseibacillus rhamnosus* HA-114 selectively improved hippocampal-dependent cognitive deficits in 6-OHDA induced PD-like symptoms in rodents [85]. Moreover, Hsieh et al. showed that long-term administration of probiotics protects dopamine neurons and mitigates the deterioration of motor dysfunctions in MitoPark PD mice [86]. Similarly, omega-3 fatty acids attenuated nigrostriatal degeneration and improved motor activity in MPTP-induced mouse models, likely through stabilization of neuronal membranes and resolution of neuroinflammation [87]. Vitamin D has demonstrated neuroprotective effects in PD models by reducing dopaminergic neuron apoptosis and suppressing inflammatory signaling [88], as well as enhancing striatal dopamine release and nigral dopamine content [89]. Moreover, Prajapati et al. demonstrated administration of coenzyme Q10 facilitates mitochondrial function and prevent 6-OHDA-induced dopaminergic toxicity in rodent [81]. Collectively, preclinical studies indicate that probiotics and nutritional supplements can modulate α-synuclein-associated pathology, oxidative stress, mitochondrial dysfunction, and dopaminergic integrity in PD-relevant models.

#### 4.2.2. Clinical Evidence

Clinical evidence most strongly supports the use of probiotics for the management of PD-associated gastrointestinal dysfunction, particularly constipation. Early clinical studies demonstrated that fermented milk containing *Lactobacillus casei* Shirota significantly increased stool frequency and improved stool consistency in constipated PD patients [90]. These findings were subsequently confirmed in larger randomized controlled trials, including a Class I study by Barichella et al. which showed that four weeks of synbiotic treatment (multi-strain probiotics combined with prebiotic fiber) significantly increased the number of complete bowel movements and improved constipation severity compared with placebo [82]. More recent trials further demonstrated that *Lacticaseibacillus paracasei* Shirota supplementation not only alleviated gastrointestinal symptoms but also favorably altered gut microbiota composition toward increased short-chain fatty acid–producing taxa [83]. Moreover, systematic reviews and meta-analyses have consistently concluded that probiotics reliably improve gastrointestinal outcomes in PD, with modest and variable effects on motor and non-motor symptoms, limited in part by study heterogeneity and small sample sizes [19].

Dietary supplements have been evaluated primarily for their systemic and non-motor benefits. Omega-3 fatty acid supplementation has shown modest improvements in mood and quality of life in PD patients, as demonstrated in randomized controlled trials [91]. In a double-blind, placebo-controlled study, omega-3 combined with vitamin E improved Unified Parkinson’s Disease Rating Scale (UPDRS) total scores, enhanced antioxidant capacity (increased total antioxidant capacity and glutathione, reduced malondialdehyde), and reduced systemic inflammation (hs-CRP) [92]. Vitamin D supplementation has been associated with improved postural stability and reduced fall risk, with the greatest benefit observed in PD patients carrying specific vitamin D receptor (VDR) genotypes [93]. In contrast, despite early promise, large phase III trials evaluating coenzyme Q10 (QE3 trial) failed to demonstrate disease-modifying efficacy, as exemplified by the negative results of the QE3 trial, which halted further development of this compound for PD [9,94].

In summary, clinical and preclinical evidence indicates that probiotics provide consistent and reproducible benefits for gastrointestinal symptoms in PD, representing the most robust application of microbiota-targeted therapy in this disease. Nutritional supplements such as omega-3 fatty acids and vitamin D may offer supportive benefits for selecting non-motor and systemic outcomes, including mood, oxidative stress, and fall prevention, but no intervention to date has demonstrated clear disease-modifying effects on PD progression.

### 4.3. Mild Cognitive Impairment and Age-Related Cognitive Decline (MCI)

MCI represents a transitional state between normal aging and dementia, with an annual conversion rate to AD of approximately 10–15%. Age-related cognitive decline (ARCD) refers to subtler, subclinical impairments in memory and executive function commonly associated with vascular dysfunction, micronutrient deficiencies, metabolic alterations, and age-related changes in gut microbiota. Given their relatively early position along the cognitive continuum, both MCI and age-related cognitive decline (ARCD) represent stages of high preventive and therapeutic potential for microbiota- and nutrition-based interventions.

#### 4.3.1. Preclinical Evidence

Preclinical studies in aging and cognitive impairment models provide strong mechanistic support for microbiome-targeted and nutritional interventions. Probiotic administration in aged rodents enhances hippocampal brain-derived neurotrophic factor (BDNF) expression and improves learning and memory performance, while microbial metabolites such as short-chain fatty acids regulate immune, epigenetic, and synaptic plasticity–related pathways [18,51,52]. Polyphenols derived from blueberries, grapes, and other plant sources reduce oxidative stress and promote neuronal resilience in aging models [71,72,95]. Omega-3 fatty acids support dendritic integrity, synaptic plasticity, and cognitive flexibility [73,74]. While B vitamins exert neuroprotective effects by lowering homocysteine levels, a key contributor to hippocampal degeneration and vascular dysfunction [56,88,96]. Together, these animal studies demonstrate that microbiota modulation and targeted nutritional support converge on pathways relevant to synaptic maintenance and cognitive resilience during aging.

#### 4.3.2. Clinical Evidence

Clinical trials in MCI populations provide consistent evidence for modest but reproducible cognitive benefits. Several randomized studies report improvements in MMSE scores, verbal learning, and memory domains following probiotic supplementation, accompanied by reductions in inflammatory and metabolic markers [77]. A systematic review further concluded that probiotics exert small but consistent benefits on cognition and mood in MCI, with outcomes influenced by strain specificity, intervention duration, and baseline risk profile [19]. B-vitamin supplementation (B6, B12, and folate) has been shown to reduce homocysteine levels and significantly slow hippocampal atrophy in magnetic resonance imaging (MRI)-based studies of individuals with MCI [56]. Polyphenol-based interventions, including resveratrol, cocoa flavanols, and berry-derived compounds, improve memory, executive function, and processing speed in small randomized controlled trials [54,58]. Omega-3 supplementation, particularly DHA-rich formulations, is associated with improved learning and memory performance in older adults with subjective cognitive decline or MCI [65,66].

Direct probiotic evidence in MCI is further supported by a randomized, double-blind, placebo-controlled trial demonstrating that 16 weeks of *Bifidobacterium breve* MCC1274 supplementation improved immediate memory and visuospatial/constructional abilities while reducing circulating inflammatory markers [76]. These findings align with broader meta-analyses indicating that probiotic interventions yield small but reliable cognitive benefits across MCI and early neurodegenerative stages [19].

Clinical data in ARCD and cognitively healthy aging populations reinforce these observations. In older adults with ARCD, resveratrol supplementation improved memory, enhanced hippocampal functional connectivity, and optimized glucose metabolism [58]. Reviews of cocoa flavanol and berry extract interventions consistently report benefits in executive function and processing speed [54]. However, omega-3 supplementation has been associated with improved memory and learning outcomes in aging cohorts [66]. Large-scale trials further support targeted supplementation strategies: in the COSMOS-Mind trial, daily multivitamin–mineral supplementation over three years significantly slowed global cognitive decline and improved episodic memory and executive function, particularly among participants with cardiovascular disease [97]. Probiotic trials in community-dwelling older adults also report improvements in mental flexibility, stress resilience, and gut microbiota composition, with broader syntheses supporting strain- and dose-dependent benefits for verbal learning, mood, and executive function [19,54,98]. Functional neuroimaging studies further demonstrate that cocoa flavanols selectively enhance dentate gyrus function and pattern-separation memory, highlighting hippocampal subregion-specific sensitivity to dietary modulation [99].

In summary, convergent preclinical and clinical evidence indicates that probiotics, B vitamins, omega-3 fatty acids, and polyphenols provide complementary and incremental cognitive benefits in MCI and ARCD. While effect sizes are modest, these interventions consistently improve memory and executive function, reduce systemic inflammation, and support hippocampal integrity. When initiated early and maintained over time, microbiota- and nutrition-based strategies may help delay progression from MCI or ARCD toward overt dementia.

**Table 1:** Representative clinical trials evaluating probiotics and dietary supplements in age-related neurodegenerative disorders.

Author (Year)	Disease/Population	Study Design	N (Number of Participants)	Intervention (Strain/Supplement)	Duration	Primary Outcomes	Key Findings
Akbari et al. (2016) [53]	AD	Randomized, double-blind, placebo-controlled	60	*Lactobacillus acidophilus*, *L. casei*, *Bifidobacterium bifidum*, *L. fermentum*	12 weeks	MMSE, metabolic markers	Modest but significant improvement in MMSE; reduced insulin resistance and inflammatory markers
Tamtaji et al. (2019) [75]	AD	Randomized, double-blind, placebo-controlled	79	Multi-strain probiotics + selenium	12 weeks	MMSE, oxidative stress, cytokines	Small improvement in MMSE; reduced IL-6, TNF-α, oxidative stress
Xiao et al. (2020) [76]	MCI	Randomized, double-blind, placebo-controlled	80	*Bifidobacterium breve* MCC1274	16 weeks	Cognitive battery, inflammatory markers	Improved memory and visuospatial function; reduced CRP and IL-1β
Naomi et al. (2021) [19]	AD & MCI (meta-analysis)	Systematic review & meta-analysis	22	Various probiotic formulations	8–24 weeks	MMSE, ADAS-Cog	Small but consistent cognitive improvements; high heterogeneity
Barichella et al. (2016) [82]	PD	Randomized, double-blind, placebo-controlled	120	Multi-strain probiotics + prebiotic fiber (synbiotic)	4 weeks	Constipation scores, GI outcomes	Significant improvement in bowel function; no disease-modifying effects
Yang et al. (2023) [83]	PD	Randomized, double-blind, placebo-controlled	56	*Lacticaseibacillus paracasei* Shirota	12 weeks	GI symptoms, microbiome composition	Improved GI symptoms; favorable microbiome shifts
Yurko-Mauro et al. (2010) [66]	Age-related cognitive decline	Randomized, double-blind, placebo-controlled	485	DHA-rich omega-3 fatty acids	24 weeks	Memory performance	Modest improvement in memory in early cognitive decline
Witte et al. (2014) [58]	Healthy older adults	Randomized, placebo-controlled	46	Resveratrol	26 weeks	Memory, hippocampal connectivity	Improved memory and hippocampal functional connectivity
Baker et al. (2023) [97]	Older adults	Randomized, placebo-controlled	>2000	Multivitamin–mineral supplement	3 years	Global cognition	Slowed cognitive decline; greater benefit in high-risk individuals

## 5. Limitations

Despite promising findings, the current literature on probiotics and dietary supplements in cognitive aging has several important limitations. Most randomized clinical trials are underpowered, enrolling small cohorts (typically 30–120 participants), which limits statistical power and generalizability [53]. Intervention durations are often short (8–24 weeks), restricting assessment of long-term cognitive trajectories, disease progression, or conversion from MCI to dementia [58,75]. Substantial heterogeneity across studies-particularly in probiotic strain composition, dosing regimens, delivery formats, and supplement formulations and bioavailability, further complicates cross-study comparisons and meta-analytic synthesis, precluding standardized clinical recommendations [16]. Few trials evaluate clinically meaningful long-term outcomes such as hippocampal atrophy rates, sustained quality-of-life improvements, or dementia incidence; for example, while the VITACOG trial demonstrated reduced brain atrophy with B-vitamin supplementation in MCI [96], most studies have follow-up periods of six months or less, and even large trials such as COSMOS-Mind did not assess dementia incidence [97]. Although multiple randomized trials and meta-analyses report statistically significant cognitive benefits, effect sizes are generally modest, heterogeneous, and short-term, with limited evidence for durable or disease-modifying effects, indicating that these interventions should be considered adjunctive rather than stand-alone therapies. Finally, while gut microbiome alterations are consistently associated with neurodegenerative disorders, causal relationships remain unclear due to largely associative human data, and although probiotics are generally well tolerated, rare cases of bacteremia and sepsis have been reported in immunocompromised individuals, underscoring the need for careful patient selection and safety monitoring.

## 6. Challenges and Future Directions

One of the major challenges in translating microbiome- and nutrition-based interventions into clinical practice is the need for strain- and nutrient-specific recommendations. Although certain probiotic strains, such as *Bifidobacterium breve* MCC1274 and *Lacticaseibacillus paracasei* Shirota, have demonstrated cognitive or gastrointestinal benefits [76,83], outcomes vary substantially depending on strain, dose, formulation, and delivery format [19,77]. Similarly, not all omega-3 fatty acids or polyphenols exert equivalent effects, with DHA showing more consistent memory benefits than EPA [66,73], and cocoa flavanols producing domain-specific improvements in executive function and hippocampal memory [99]. These findings underscore the need to move beyond broad intervention categories toward precision nutrition frameworks that account for host-specific factors, including baseline gut microbiota composition, habitual diet, and genetic background. Differential responses to supplementation have already been observed based on vitamin D receptor genotype in PD [93] and *APOE* genotype in AD risk modulation with omega-3 fatty acids [65]. Advances in microbiome sequencing, metabolomics, and bioinformatics now enable stratification of individuals into predicted responders and non-responders, thereby reducing trial heterogeneity and improving translational precision [18,24]. Emerging evidence also supports combined and multimodal strategies, including synbiotics, which have improved constipation and quality of life in PD [82], postbiotics such as short-chain fatty acids and indole derivatives that offer more stable pharmacological effects [25,26], and multi-nutrient formulations such as Souvenaid and LipiDiDiet, which have shown benefits in prodromal AD [55,100]. Integration of multi-omics approaches—including metagenomics, metabolomics, proteomics, immune phenotyping, and host genomics—represents a critical step toward mechanistic resolution and precision-guided intervention design, as these datasets capture microbial–metabolic–immune networks implicated in neurodegeneration [7,18,24,25,47,64]. Systems-level bioinformatics, network analysis, and machine-learning models have demonstrated promise in identifying microbial–host signatures predictive of disease progression and treatment responsiveness [78,97]. The application of artificial intelligence further enables modeling of complex, nonlinear interactions across microbiome, diet, metabolism, genetics, and clinical outcomes, facilitating optimized strain selection, nutrient combinations, dosing strategies, and biomarker-enriched trial design [18,24,78]. Collectively, integration of multi-omics, computational modeling, and AI-driven analytics is essential for improving reproducibility, minimizing heterogeneity, and advancing microbiome-based and nutritional interventions toward clinically actionable, personalized strategies for aging and neurodegenerative diseases

## 7. Conclusions

Recent scientific discoveries have generated significant preclinical and clinical data that indicate probiotics and dietary supplements hold substantial promise for combating cognitive aging and deterioration. In both preclinical models and human studies, a variety of probiotic strains, omega-3 fatty acids, B vitamins, and polyphenols have been shown to have positive effects on synaptic plasticity and resilience, neuroinflammation, and metabolic balance. At the clinical level, these supplements have been associated with modest but meaningful improvements in cognition and well-being, indicating their translational potential. Evidence from randomized trials and meta-analyses in AD, PD, and MCI is the most advanced and shows consistently reproducible albeit small effects of these interventions on commonly used cognitive outcomes (MMSE, ADAS-Cog, RBANS) in these conditions. Strain- and nutrient-specific effects on cognition, modulation by host and clinical characteristics, and limited generalizability to other settings due to small sample sizes and short durations are common across these studies, highlighting an important need for large, multicenter, adequately powered, and longer-term follow-up trials to determine if and how these approaches might influence disease trajectories and conversion to dementia. In the future, manipulation of the gut–brain axis and nutritional deficiencies may emerge as important adjunct therapeutic targets for age-related neurodegenerative diseases and brain aging. The development of multimodal interventions that combine pre- and probiotics, synbiotics, and postbiotics with current evidence-based approaches in nutrient-based interventions (multivitamins, omega-3s, B vitamins) has the potential to be safe, scalable, and individually targeted.

## Data Availability

No new data were created or analyzed in this study. Data sharing is not applicable.
